# (2*E*,2′*E*)-1,1′-([1,1′-Biphen­yl]-4,4′-di­yl)bis­[3-(di­meth­yl­amino)­prop-2-en-1-one]

**DOI:** 10.1107/S2414314624003584

**Published:** 2024-04-26

**Authors:** Tomohiro Minagawa, Masaaki Sadakiyo

**Affiliations:** a Department of Applied Chemistry, Faculty of Science Division I, Tokyo University, of Science, 1-3 Kagurazaka, Shinjuku-ku, Tokyo, 162-8601, Japan; University of Antofagasta, Chile

**Keywords:** crystal structure, *β*-keto­amine, π-conjugated mol­ecule

## Abstract

The title compound, C_22_H_24_N_2_O_2_, crystallizes in space group *P*2_1_/*n*. The mol­ecular structure is almost planar except for a tilt of the phenyl rings. The allyl groups on both ends exhibit the *trans*-form and the connected N atoms show *sp* ^2^ character. The mol­ecules are stacked and assembled along the *c*-axis direction by C—H⋯π inter­actions.

## Abstract 2

The π-conjugated planar title mol­ecules are assembled through C—H⋯π inter­actions between neighboring mol­ecules, resulting in stacking along the *c*-axis.

## Graphical abstract

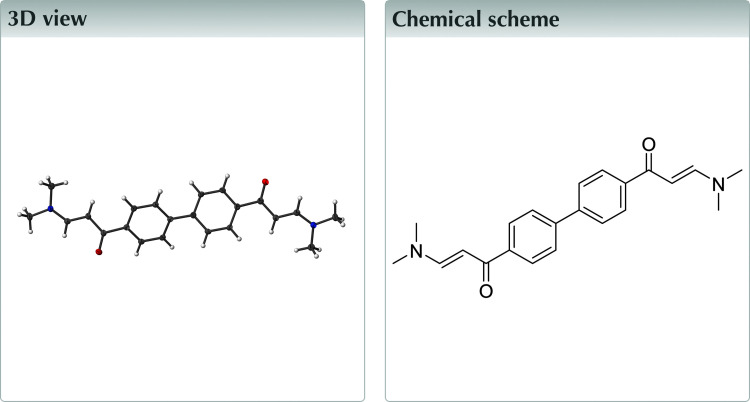

## Structure description

**Figure 1:**
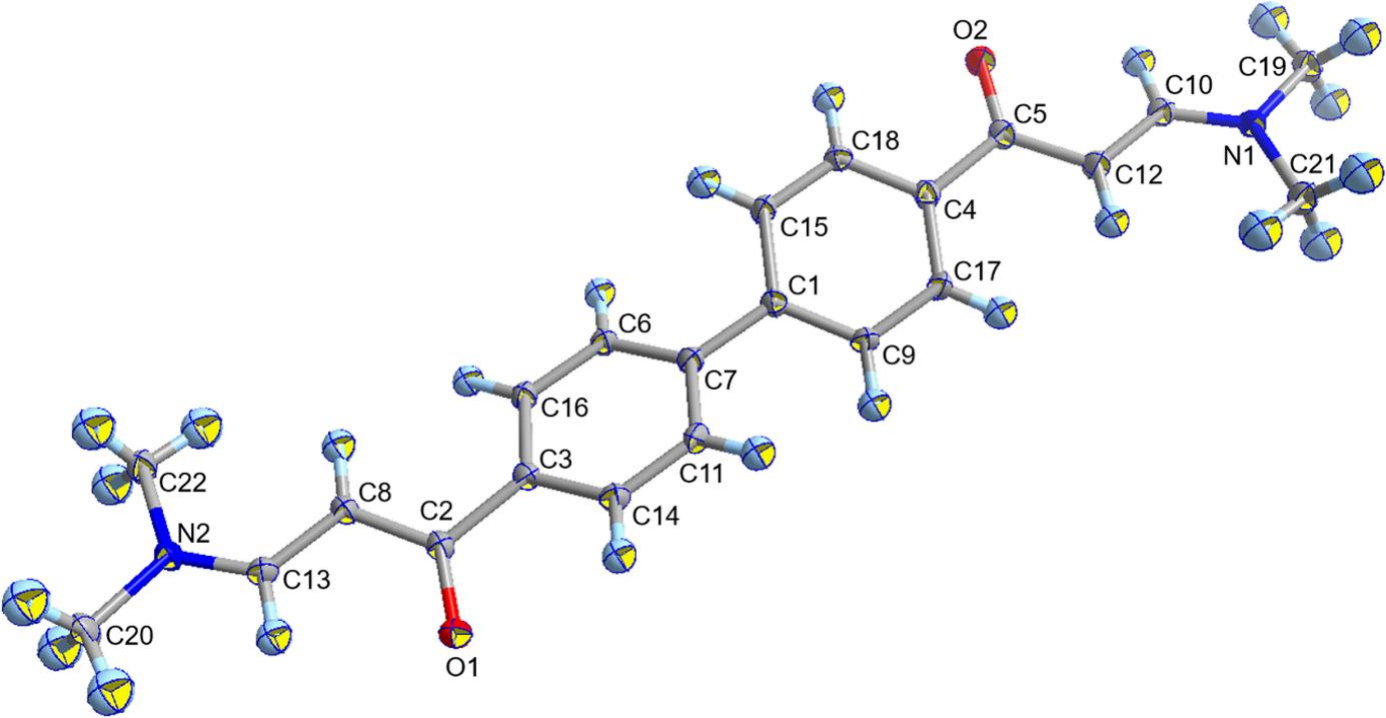
Illustration of the title mol­ecule, composed of crystallographically independent atoms with displacement ellipsoids drawn at the 50% probability level.

*β*-Keto­amines are important not only for various chemical reactions, but also for creating functional complexes (Pettinari *et al.*, 2014 ▸). Recently, they have also been used as reagents for covalent organic frameworks (Zhao *et al.*, 2023 ▸). In this work, the crystal structure of the title *β*-keto­amine was determined. The mol­ecule is almost flat, but the phenyl rings are tilted [dihedral angle = 30.14 (8)°] (Fig. 1 ▸) as a result of the repulsion between H atoms on the phenyl rings and inter­molecular inter­actions, *i.e.*, C—H⋯π, with neighboring mol­ecules (Table 1 ▸). In addition to the carbonyl and the allyl groups, the amino groups also show a planar character, indicating the *sp* ^2^ character of the N atoms and the π-conjugated character of these functional groups. The bond-angle sums for both N atoms are 360.0°.

**Figure 2:**
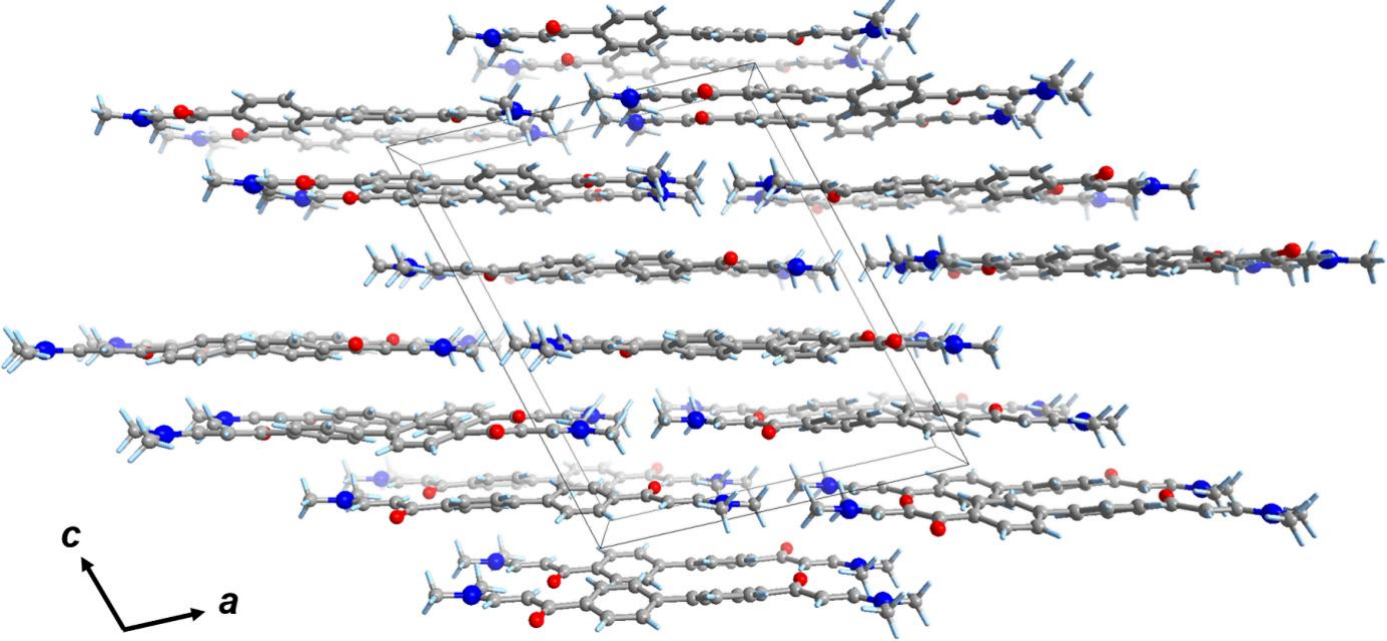
Packing structure of the title compound along the *b* axis.

**Figure 3:**
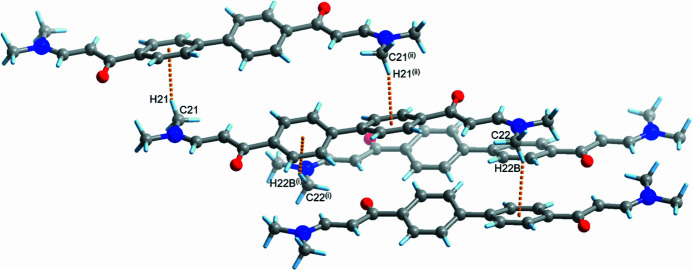
C—H⋯π inter­actions between neighboring mol­ecules. Symmetry codes: (i) 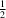 − *x*, 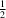 + *y*, 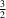 − *z*; (ii) −*x*, −*y*, 2 − *z*.

In the crystal, the mol­ecules are assembled to form a two-dimensional layer-like structure in the (105) plane (Fig. 2 ▸). The mol­ecules are stacked perpendicular to this plane through C—H⋯π inter­actions. Each mol­ecule inter­acts with three neighboring mol­ecules. Two different phenyl groups on the mol­ecule accept the C donors (C21 and C22), resulting in additional C—H⋯π inter­actions (Tsuzuki *et al.*, 2000 ▸) with mol­ecules above and below (Fig. 3 ▸). The C21 methyl group on one side inter­acts with the phenyl ring of the stacked mol­ecule above through a C21—H21⋯*Cg*2 inter­action [C21⋯*Cg2* = 3.538 (2) Å], while the C22 methyl group on the other side similarly inter­acts with the mol­ecule below [C22⋯*Cg*1 = 3.608 (2) Å] where *Cg*1 and *Cg*2 are the centroids of the C1/C9/C17/C4/C18/C15 and C3/C14/C11/C7/C6/C16 rings, respectively. The distances between the phenyl groups are not remarkably short with centroid–centroid separations of 4.363 (1) and 4.833 (1) Å, and no obvious π–π inter­actions occur with neighboring mol­ecules, indicating that the mol­ecules are assembled mainly through C—H⋯π inter­actions. C—H⋯O inter­actions involving H22 and H22*B* (Table 1 ▸) also occur, which also contribute to assemble the molecules.

## Synthesis and crystallization

A mixture of 4,4′-diacetyl biphenyl (0.953 g, 4.00 mmol), anhydrous DMF (12 ml), and *N*,*N*-di­methyl­formamide diethyl acetal (12 ml) was stirred and heated at 90°C under a nitro­gen atmosphere for 12 h. After cooling, diethyl ether (20 ml) was added slowly to the reaction mixture, resulting in a yellow powder. The precipitate was collected by suction filtration and it was then immersed in *n*-pentane for 4 h at room temperature. After that, the precipitate was collected by suction filtration and dried under vacuum at 170°C overnight (0.961 g, 1.98 mmol, yield 50%). Crystals of the title compound were obtained by recrystallization through slow evaporation of a methanol solution. After several days, yellow crystals were obtained.

## Refinement

Details of crystal data, data collections, and structure refinements are shown in Table 2 ▸.

## Supplementary Material

Crystal structure: contains datablock(s) I. DOI: 10.1107/S2414314624003584/bx4027sup1.cif

Structure factors: contains datablock(s) I. DOI: 10.1107/S2414314624003584/bx4027Isup2.hkl

Supporting information file. DOI: 10.1107/S2414314624003584/bx4027Isup3.cdx

Supporting information file. DOI: 10.1107/S2414314624003584/bx4027Isup4.cml

CCDC reference: 2349722

Additional supporting information:  crystallographic information; 3D view; checkCIF report

## Figures and Tables

**Table 1:** Hydrogen-bond geometry (Å, °) *Cg*1 and *Cg*2 are the centroids of the C1/C9/C17/C4/C18/C15 and C3/C14/C11/C7/C6/C16 rings, respectively.

*D*—H⋯*A*	*D*—H	H⋯*A*	*D*⋯*A*	*D*—H⋯*A*
C21—H21*B*⋯O2^i^	0.98	2.51	3.410 (2)	152
C22—H22⋯O1^ii^	0.98	2.48	3.387 (2)	154
C21—H21⋯*Cg*2^iii^	0.98	2.60	3.538 (2)	160
C22—H22*B*⋯*Cg*1^iv^	0.98	2.70	3.608 (2)	154

Symmetry codes: (i) 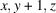 ; (ii) 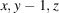 ; (iii) 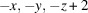 ; (iv) 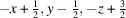 .

**Table 2:** Experimental details

Crystal data	
Chemical formula	C_22_H_24_N_2_O_2_
*M*_r_	348.43
Crystal system, space group	Monoclinic, *P*2_1_/*n*
Temperature (K)	90
*a*, *b*, *c* (Å)	15.862 (1), 6.0503 (4), 19.0640 (12)
β (°)	105.287 (3)
*V* (Å^3^)	1764.8 (2)
*Z*	4
Radiation type	Mo *K*α
μ (mm^−1^)	0.08
Crystal size (mm)	0.40 × 0.20 × 0.20

Data collection	
Diffractometer	Bruker PHOTON II CPAD
Absorption correction	Multi-scan (*SADABS*; Krause *et al.*, 2015 ▸)
*T*_min_, *T*_max_	0.669, 0.746
No. of measured, independent and observed [*I* > 2σ(*I*)] reflections	21542, 4738, 3539
*R*_int_	0.076
(sin θ/λ)_max_ (Å^−1^)	0.731

Refinement	
*R*[*F* ^2^ > 2σ(*F* ^2^)], *wR*(*F* ^2^), *S*	0.063, 0.154, 1.08
No. of reflections	4738
No. of parameters	239
H-atom treatment	H-atom parameters constrained
Δρ_max_, Δρ_min_ (e Å^−3^)	0.31, −0.25

Computer programs: *APEX4* and *SAINT* (Bruker, 2021 ▸), *SIR2019* (Burla *et al.*, 2015 ▸), *SHELXL2018/3* (Sheldrick, 2015 ▸), *DIAMOND* (Brandenburg, 2014 ▸) and *Yadokari-XG* (Kabuto *et al.*, 2009 ▸).

## full crystallographic data

### Crystal data

**Table:**

C_22_H_24_N_2_O_2_	*F*(000) = 744
*M**_r_* = 348.43	*D*_x_ = 1.311 Mg m^−^^3^
Monoclinic, *P*2_1_/*n*	Mo *K*α radiation, λ = 0.71069 Å
*a* = 15.862 (1) Å	Cell parameters from 2411 reflections
*b* = 6.0503 (4) Å	θ = 3.0–29.0°
*c* = 19.0640 (12) Å	µ = 0.08 mm^−^^1^
β = 105.287 (3)°	*T* = 90 K
*V* = 1764.8 (2) Å^3^	Block, yellow
*Z* = 4	0.40 × 0.20 × 0.20 mm

### Data collection

**Table:**

Bruker PHOTON II CPAD diffractometer	3539 reflections with *I* > 2σ(*I*)
Radiation source: fine-focus sealed tube	*R*_int_ = 0.076
φ and ω scans	θ_max_ = 31.3°, θ_min_ = 1.9°
Absorption correction: multi-scan (SADABS; Krause *et al.*, 2015)	*h* = −22→23
*T*_min_ = 0.669, *T*_max_ = 0.746	*k* = −8→8
21542 measured reflections	*l* = −27→26
4738 independent reflections	

### Refinement

**Table:**

Refinement on *F*^2^	0 restraints
Least-squares matrix: full	Hydrogen site location: inferred from neighbouring sites
*R*[*F*^2^ > 2σ(*F*^2^)] = 0.063	H-atom parameters constrained
*wR*(*F*^2^) = 0.154	*w* = 1/[σ^2^(*F*_o_^2^) + (0.0561*P*)^2^ + 0.8647*P*] where *P* = (*F*_o_^2^ + 2*F*_c_^2^)/3
*S* = 1.08	(Δ/σ)_max_ < 0.001
4738 reflections	Δρ_max_ = 0.31 e Å^−^^3^
239 parameters	Δρ_min_ = −0.25 e Å^−^^3^

### Special details

**Table:**

Geometry. All esds (except the esd in the dihedral angle between two l.s. planes) are estimated using the full covariance matrix. The cell esds are taken into account individually in the estimation of esds in distances, angles and torsion angles; correlations between esds in cell parameters are only used when they are defined by crystal symmetry. An approximate (isotropic) treatment of cell esds is used for estimating esds involving l.s. planes.
Refinement. Refinement of F^2^ against ALL reflections. The weighted R-factor wR and goodness of fit S are based on F^2^, conventional R-factors R are based on F, with F set to zero for negative F^2^. The threshold expression of F^2^ > 2sigma(F^2^) is used only for calculating R-factors(gt) etc. and is not relevant to the choice of reflections for refinement. R-factors based on F^2^ are statistically about twice as large as those based on F, and R- factors based on ALL data will be even larger. All hydrogen atoms were fixed geometrically and refined using a riding-model approximation with C—H = 0.95 (for phenyl and allyl) and 0.98 (for methyl) Å.

### Fractional atomic coordinates and isotropic or equivalent isotropic displacement parameters (Å^2^)

**Table:**

	*x*	*y*	*z*	*U*_iso_*/*U*_eq_	
O1	0.44366 (8)	0.0656 (2)	0.82473 (8)	0.0251 (3)	
O2	−0.18571 (8)	−0.6345 (2)	0.92406 (8)	0.0260 (3)	
C1	0.08648 (11)	−0.3027 (3)	0.88219 (9)	0.0172 (3)	
C2	0.42078 (11)	−0.1318 (3)	0.82155 (9)	0.0183 (4)	
N1	−0.35165 (9)	−0.1752 (2)	0.97342 (8)	0.0206 (3)	
N2	0.60934 (9)	−0.3981 (3)	0.77673 (9)	0.0216 (3)	
C3	0.33358 (11)	−0.1860 (3)	0.83472 (9)	0.0164 (3)	
C4	−0.07530 (11)	−0.3830 (3)	0.91419 (9)	0.0182 (4)	
C5	−0.16200 (11)	−0.4379 (3)	0.92887 (10)	0.0194 (4)	
C6	0.23300 (11)	−0.4325 (3)	0.87142 (9)	0.0184 (4)	
H6	0.220473	−0.575129	0.886905	0.022*	
C7	0.17109 (11)	−0.2637 (3)	0.86495 (9)	0.0171 (4)	
C8	0.47315 (11)	−0.3113 (3)	0.80669 (10)	0.0190 (4)	
H8	0.454442	−0.460179	0.807627	0.023*	
C9	0.04331 (11)	−0.1358 (3)	0.90959 (10)	0.0194 (4)	
H9	0.069054	0.006774	0.918150	0.023*	
C10	−0.29110 (11)	−0.3106 (3)	0.96087 (9)	0.0197 (4)	
H10	−0.302961	−0.463891	0.963229	0.024*	
C11	0.19194 (11)	−0.0561 (3)	0.84176 (10)	0.0188 (4)	
H11	0.150491	0.060452	0.835705	0.023*	
C12	−0.21424 (11)	−0.2595 (3)	0.94506 (10)	0.0204 (4)	
H12	−0.195669	−0.110271	0.944727	0.024*	
C13	0.55029 (11)	−0.2624 (3)	0.79123 (10)	0.0197 (4)	
H13	0.563685	−0.109429	0.790785	0.024*	
C14	0.27188 (11)	−0.0173 (3)	0.82748 (9)	0.0182 (4)	
H14	0.284837	0.125692	0.812585	0.022*	
C15	0.04694 (11)	−0.5114 (3)	0.87134 (10)	0.0206 (4)	
H15	0.074488	−0.627754	0.852269	0.025*	
C16	0.31225 (11)	−0.3953 (3)	0.85566 (9)	0.0176 (4)	
H16	0.352524	−0.513573	0.859146	0.021*	
C17	−0.03656 (11)	−0.1741 (3)	0.92460 (10)	0.0197 (4)	
H17	−0.065153	−0.056678	0.942160	0.024*	
C18	−0.03162 (11)	−0.5510 (3)	0.88793 (10)	0.0193 (4)	
H18	−0.056200	−0.695105	0.881307	0.023*	
C19	−0.42660 (11)	−0.2587 (3)	0.99574 (10)	0.0231 (4)	
H19	−0.425634	−0.420614	0.995747	0.035*	
H19A	−0.480571	−0.206431	0.961739	0.035*	
H19B	−0.424069	−0.205172	1.044775	0.035*	
C20	0.68514 (12)	−0.3137 (3)	0.75587 (11)	0.0259 (4)	
H20	0.683659	−0.151744	0.755483	0.039*	
H20A	0.684187	−0.367920	0.707229	0.039*	
H20B	0.738614	−0.364478	0.790844	0.039*	
C21	−0.34611 (12)	0.0646 (3)	0.96831 (11)	0.0248 (4)	
H21	−0.334516	0.129235	1.017057	0.037*	
H21A	−0.401469	0.122254	0.937915	0.037*	
H21B	−0.298604	0.103377	0.946522	0.037*	
C22	0.60316 (12)	−0.6375 (3)	0.78032 (11)	0.0239 (4)	
H22	0.553718	−0.677418	0.799595	0.036*	
H22A	0.657287	−0.696557	0.812301	0.036*	
H22B	0.594317	−0.699911	0.731468	0.036*	

### Atomic displacement parameters (Å^2^)

**Table:**

	*U*^11^	*U*^22^	*U*^33^	*U*^12^	*U*^13^	*U*^23^
O1	0.0229 (6)	0.0164 (6)	0.0391 (8)	−0.0025 (5)	0.0138 (6)	−0.0007 (6)
O2	0.0243 (7)	0.0191 (7)	0.0391 (8)	−0.0041 (5)	0.0161 (6)	−0.0028 (6)
C1	0.0160 (8)	0.0166 (8)	0.0198 (8)	0.0012 (7)	0.0060 (7)	0.0014 (7)
C2	0.0168 (8)	0.0191 (9)	0.0206 (8)	−0.0004 (7)	0.0076 (7)	−0.0003 (7)
N1	0.0188 (7)	0.0179 (8)	0.0273 (8)	−0.0018 (6)	0.0098 (6)	−0.0017 (6)
N2	0.0194 (7)	0.0180 (8)	0.0314 (8)	−0.0005 (6)	0.0135 (7)	0.0007 (6)
C3	0.0169 (8)	0.0160 (8)	0.0171 (8)	−0.0007 (7)	0.0057 (7)	−0.0006 (7)
C4	0.0178 (8)	0.0183 (9)	0.0201 (8)	0.0005 (7)	0.0075 (7)	0.0015 (7)
C5	0.0203 (8)	0.0194 (9)	0.0201 (8)	−0.0007 (7)	0.0083 (7)	0.0004 (7)
C6	0.0189 (8)	0.0141 (8)	0.0240 (9)	0.0003 (7)	0.0089 (7)	0.0005 (7)
C7	0.0186 (8)	0.0147 (8)	0.0194 (8)	−0.0002 (7)	0.0073 (7)	−0.0010 (7)
C8	0.0170 (8)	0.0187 (9)	0.0237 (9)	−0.0014 (7)	0.0094 (7)	−0.0001 (7)
C9	0.0205 (8)	0.0141 (8)	0.0251 (9)	−0.0024 (7)	0.0085 (7)	−0.0018 (7)
C10	0.0207 (8)	0.0176 (9)	0.0216 (9)	−0.0004 (7)	0.0068 (7)	−0.0006 (7)
C11	0.0166 (8)	0.0151 (8)	0.0250 (9)	0.0023 (7)	0.0056 (7)	−0.0012 (7)
C12	0.0210 (8)	0.0171 (9)	0.0257 (9)	−0.0017 (7)	0.0109 (7)	−0.0003 (7)
C13	0.0204 (8)	0.0158 (8)	0.0246 (9)	−0.0007 (7)	0.0087 (7)	0.0000 (7)
C14	0.0198 (8)	0.0145 (8)	0.0212 (8)	−0.0002 (7)	0.0071 (7)	0.0010 (7)
C15	0.0209 (8)	0.0151 (8)	0.0284 (9)	0.0016 (7)	0.0110 (8)	−0.0020 (7)
C16	0.0171 (8)	0.0155 (8)	0.0210 (8)	0.0022 (7)	0.0065 (7)	0.0003 (7)
C17	0.0208 (8)	0.0166 (9)	0.0248 (9)	−0.0003 (7)	0.0117 (7)	−0.0043 (7)
C18	0.0180 (8)	0.0143 (8)	0.0259 (9)	−0.0016 (7)	0.0064 (7)	0.0004 (7)
C19	0.0194 (8)	0.0239 (10)	0.0292 (10)	−0.0010 (7)	0.0120 (8)	0.0022 (8)
C20	0.0203 (9)	0.0246 (10)	0.0375 (11)	−0.0010 (8)	0.0160 (8)	0.0029 (8)
C21	0.0267 (9)	0.0192 (9)	0.0315 (10)	0.0013 (8)	0.0132 (8)	0.0011 (8)
C22	0.0237 (9)	0.0188 (9)	0.0324 (10)	0.0022 (7)	0.0132 (8)	0.0009 (8)

### Geometric parameters (Å, º)

**Table:**

O1—C2	1.245 (2)	C9—H9	0.9500
O2—C5	1.243 (2)	C10—C12	1.366 (2)
C1—C9	1.396 (2)	C10—H10	0.9500
C1—C15	1.401 (2)	C11—C14	1.385 (2)
C1—C7	1.482 (2)	C11—H11	0.9500
C2—C8	1.440 (2)	C12—H12	0.9500
C2—C3	1.506 (2)	C13—H13	0.9500
N1—C10	1.331 (2)	C14—H14	0.9500
N1—C19	1.455 (2)	C15—C18	1.385 (2)
N1—C21	1.458 (2)	C15—H15	0.9500
N2—C13	1.329 (2)	C16—H16	0.9500
N2—C22	1.455 (2)	C17—H17	0.9500
N2—C20	1.455 (2)	C18—H18	0.9500
C3—C14	1.395 (2)	C19—H19	0.9800
C3—C16	1.396 (2)	C19—H19A	0.9800
C4—C18	1.395 (2)	C19—H19B	0.9800
C4—C17	1.397 (2)	C20—H20	0.9800
C4—C5	1.511 (2)	C20—H20A	0.9800
C5—C12	1.443 (2)	C20—H20B	0.9800
C6—C16	1.386 (2)	C21—H21	0.9800
C6—C7	1.399 (2)	C21—H21A	0.9800
C6—H6	0.9500	C21—H21B	0.9800
C7—C11	1.400 (2)	C22—H22	0.9800
C8—C13	1.364 (2)	C22—H22A	0.9800
C8—H8	0.9500	C22—H22B	0.9800
C9—C17	1.390 (2)		
			
C9—C1—C15	117.55 (15)	N2—C13—C8	129.23 (17)
C9—C1—C7	122.00 (16)	N2—C13—H13	115.4
C15—C1—C7	120.45 (15)	C8—C13—H13	115.4
O1—C2—C8	123.86 (15)	C11—C14—C3	120.76 (16)
O1—C2—C3	118.00 (15)	C11—C14—H14	119.6
C8—C2—C3	118.14 (15)	C3—C14—H14	119.6
C10—N1—C19	121.53 (15)	C18—C15—C1	121.12 (16)
C10—N1—C21	122.76 (15)	C18—C15—H15	119.4
C19—N1—C21	115.68 (14)	C1—C15—H15	119.4
C13—N2—C22	123.09 (15)	C6—C16—C3	120.76 (16)
C13—N2—C20	121.23 (15)	C6—C16—H16	119.6
C22—N2—C20	115.68 (15)	C3—C16—H16	119.6
C14—C3—C16	118.36 (15)	C9—C17—C4	120.82 (16)
C14—C3—C2	118.40 (15)	C9—C17—H17	119.6
C16—C3—C2	123.21 (15)	C4—C17—H17	119.6
C18—C4—C17	117.95 (15)	C15—C18—C4	121.20 (16)
C18—C4—C5	117.96 (16)	C15—C18—H18	119.4
C17—C4—C5	124.09 (15)	C4—C18—H18	119.4
O2—C5—C12	123.49 (16)	N1—C19—H19	109.5
O2—C5—C4	117.97 (16)	N1—C19—H19A	109.5
C12—C5—C4	118.50 (15)	H19—C19—H19A	109.5
C16—C6—C7	121.20 (16)	N1—C19—H19B	109.5
C16—C6—H6	119.4	H19—C19—H19B	109.5
C7—C6—H6	119.4	H19A—C19—H19B	109.5
C6—C7—C11	117.64 (15)	N2—C20—H20	109.5
C6—C7—C1	121.32 (15)	N2—C20—H20A	109.5
C11—C7—C1	121.04 (15)	H20—C20—H20A	109.5
C13—C8—C2	118.39 (16)	N2—C20—H20B	109.5
C13—C8—H8	120.8	H20—C20—H20B	109.5
C2—C8—H8	120.8	H20A—C20—H20B	109.5
C17—C9—C1	121.34 (16)	N1—C21—H21	109.5
C17—C9—H9	119.3	N1—C21—H21A	109.5
C1—C9—H9	119.3	H21—C21—H21A	109.5
N1—C10—C12	128.94 (17)	N1—C21—H21B	109.5
N1—C10—H10	115.5	H21—C21—H21B	109.5
C12—C10—H10	115.5	H21A—C21—H21B	109.5
C14—C11—C7	121.23 (16)	N2—C22—H22	109.5
C14—C11—H11	119.4	N2—C22—H22A	109.5
C7—C11—H11	119.4	H22—C22—H22A	109.5
C10—C12—C5	118.29 (16)	N2—C22—H22B	109.5
C10—C12—H12	120.9	H22—C22—H22B	109.5
C5—C12—H12	120.9	H22A—C22—H22B	109.5
			
O1—C2—C3—C14	19.5 (2)	C1—C7—C11—C14	178.24 (16)
C8—C2—C3—C14	−161.06 (16)	N1—C10—C12—C5	−175.95 (17)
O1—C2—C3—C16	−158.78 (17)	O2—C5—C12—C10	4.7 (3)
C8—C2—C3—C16	20.7 (3)	C4—C5—C12—C10	−177.69 (16)
C18—C4—C5—O2	7.3 (2)	C22—N2—C13—C8	−4.7 (3)
C17—C4—C5—O2	−173.35 (18)	C20—N2—C13—C8	175.17 (19)
C18—C4—C5—C12	−170.47 (16)	C2—C8—C13—N2	179.18 (18)
C17—C4—C5—C12	8.9 (3)	C7—C11—C14—C3	1.0 (3)
C16—C6—C7—C11	0.2 (3)	C16—C3—C14—C11	0.9 (3)
C16—C6—C7—C1	−179.64 (16)	C2—C3—C14—C11	−177.45 (16)
C9—C1—C7—C6	149.01 (17)	C9—C1—C15—C18	−0.7 (3)
C15—C1—C7—C6	−30.9 (3)	C7—C1—C15—C18	179.29 (16)
C9—C1—C7—C11	−30.8 (3)	C7—C6—C16—C3	1.8 (3)
C15—C1—C7—C11	149.25 (18)	C14—C3—C16—C6	−2.3 (3)
O1—C2—C8—C13	−3.7 (3)	C2—C3—C16—C6	175.97 (16)
C3—C2—C8—C13	176.87 (16)	C1—C9—C17—C4	1.4 (3)
C15—C1—C9—C17	−0.9 (3)	C18—C4—C17—C9	−0.4 (3)
C7—C1—C9—C17	179.15 (16)	C5—C4—C17—C9	−179.73 (16)
C19—N1—C10—C12	−174.60 (18)	C1—C15—C18—C4	1.7 (3)
C21—N1—C10—C12	3.2 (3)	C17—C4—C18—C15	−1.2 (3)
C6—C7—C11—C14	−1.6 (3)	C5—C4—C18—C15	178.21 (16)

### Hydrogen-bond geometry (Å, º)

*Cg*1 and *Cg*2 are the centroids of the C1/C9/C17/C4/C18/C15 and C3/C14/C11/C7/C6/C16 rings, respectively.

**Table:**

*D*—H···*A*	*D*—H	H···*A*	*D*···*A*	*D*—H···*A*
C21—H21*B*···O2^i^	0.98	2.51	3.410 (2)	152
C22—H22···O1^ii^	0.98	2.48	3.387 (2)	154
C21—H21···*Cg*2^iii^	0.98	2.60	3.538 (2)	160
C22—H22*B*···*Cg*1^iv^	0.98	2.70	3.608 (2)	154

Symmetry codes: (i) *x*, *y*+1, *z*; (ii) *x*, *y*−1, *z*; (iii) −*x*, −*y*, −*z*+2; (iv) −*x*+1/2, *y*−1/2, −*z*+3/2.
